# Automatic capture management may cause unnecessary battery depletion in selective his‐bundle pacing

**DOI:** 10.1002/ccr3.3168

**Published:** 2020-07-23

**Authors:** Hung‐Pin Wu, Jan‐Yow Chen, Kuo‐Hung Lin, Kuan‐Cheng Chang

**Affiliations:** ^1^ Division of Cardiovascular Medicine Department of Medicine China Medical University Hospital Taichung Taiwan; ^2^ School of Medicine China Medical University Taichung Taiwan

**Keywords:** capture management, device longevity, his‐bundle pacing

## Abstract

A routine change in the automatic capture management algorithm from “adaptive” to “off or monitor” is required to conserve device longevity in a permanent pacemaker with His‐bundle pacing.

## Graphical abstract

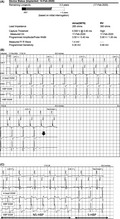

## 1 INTRODUCTION

A modern implantable pulse generator is equipped with an automatic capture management (ACM) program that provides reliable pacing threshold management and potential device longevity benefits. However, an accurate His‐bundle pacing (HBP) lead threshold value does not always correspond with the ACM algorithm measurement. We report a patient who presented with a high ventricular pacing output that reduced device longevity as a result of erroneous ACM algorithm HBP threshold measurement. Because of the time interval between pacing stimulation and the ventricular electrogram during HBP, the ACM algorithm considers “pacing capture loss” despite His‐bundle capture. The ACM algorithm overestimates an accurate HBP threshold and unnecessarily changes pacing parameters, resulting in a high ventricular pacing output. A routine change in the ACM algorithm from “adaptive” to “off or monitor” is required to conserve device longevity.

The number of patients with permanent His‐bundle pacing (HBP) has been increasing due to the feasibility of the method and its association with improved exercise capacity, ventricular synchrony, and better clinical outcomes compared with right ventricular pacing (RVP).^1^, ^2^ However, at present, no implantable pulse generator (IPG) is available with a dedicated His‐pacing port. Special device programming is required for HBP because of its unique electrical parameters.^3^, ^4^ For example, most modern IPGs are equipped with automatic capture management (ACM) programs that provide reliable pacing threshold management and potential device longevity benefits.^5^, ^6^ However, accurate HBP lead threshold values are not always the same as the ACM algorithm measurements. Here, we report a patient who presented with a relatively high ventricular pacing output that reduced device longevity as a result of an incorrect ACM algorithm HBP threshold measurement.

## 2 CASE PRESENTATION

**FIGURE 1:**
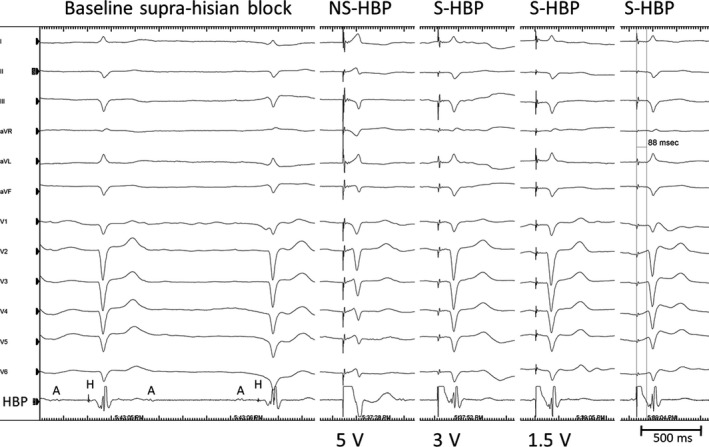
The 12‐lead electrocardiogram and intracardiac electrogram obtained from the His‐bundle pacing (HBP) lead. (Left) Baseline sinus rhythm with suprahisian block. (Right) With pacing at 5 V at 0.4 ms, nonselective HBP is noted with the culmination of both His‐bundle and ventricular capture. With pacing at 1.5‐3.0 V at 0.4 ms, selective HBP is noted with a pacing stimulus to the surface QRS onset interval of 88 ms

**FIGURE 2:**
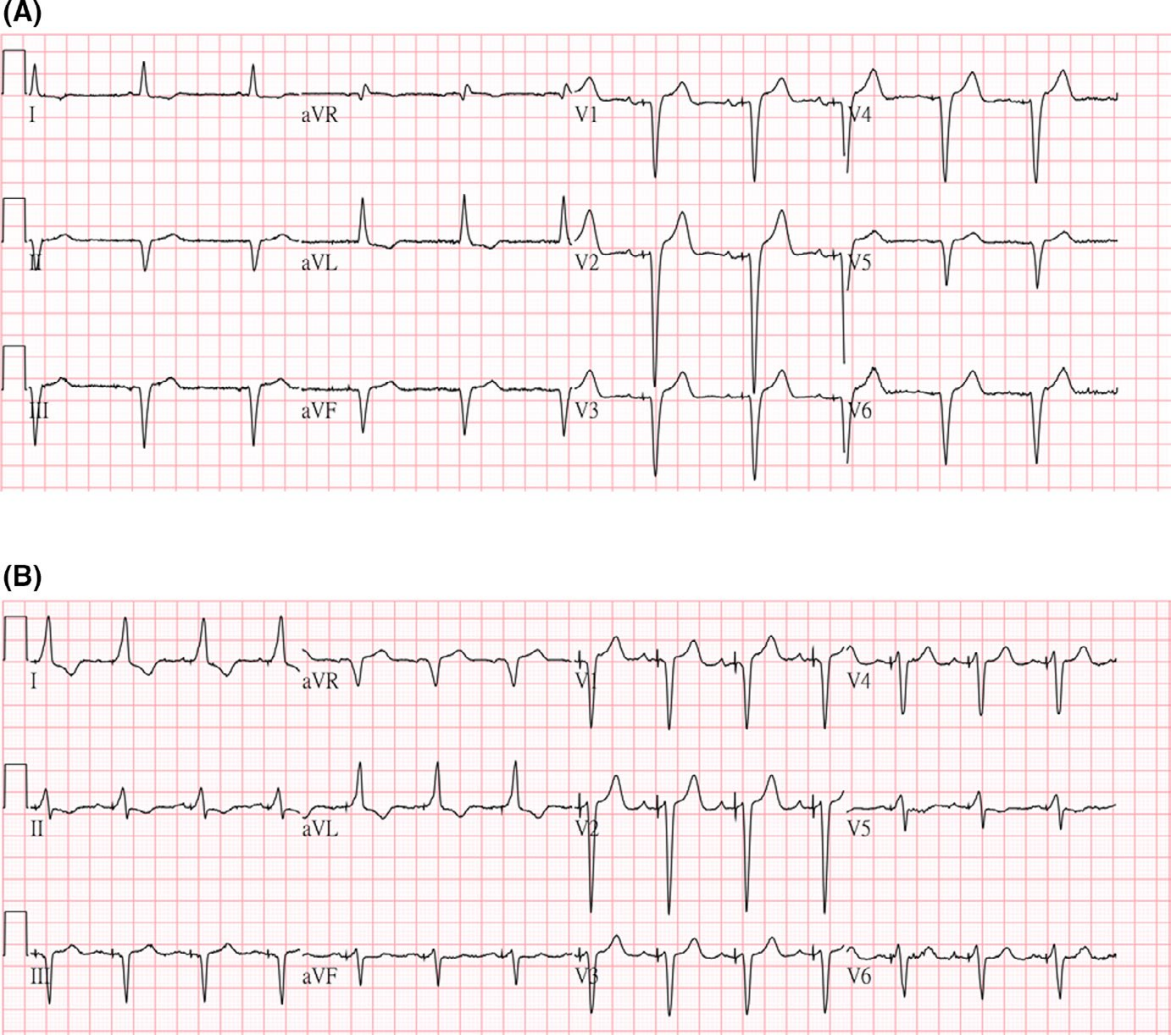
A, The 12‐lead electrocardiogram (ECG) immediately after operation. The paced QRS morphology is the same as the native QRS morphology with a pacing stimulus to the QRS onset time delay. B, The 12‐lead ECG on postoperation day 1. The paced QRS morphology is wider than the native QRS morphology, with a pseudodelta wave occurring due to local ventricular capture

**FIGURE 3:**
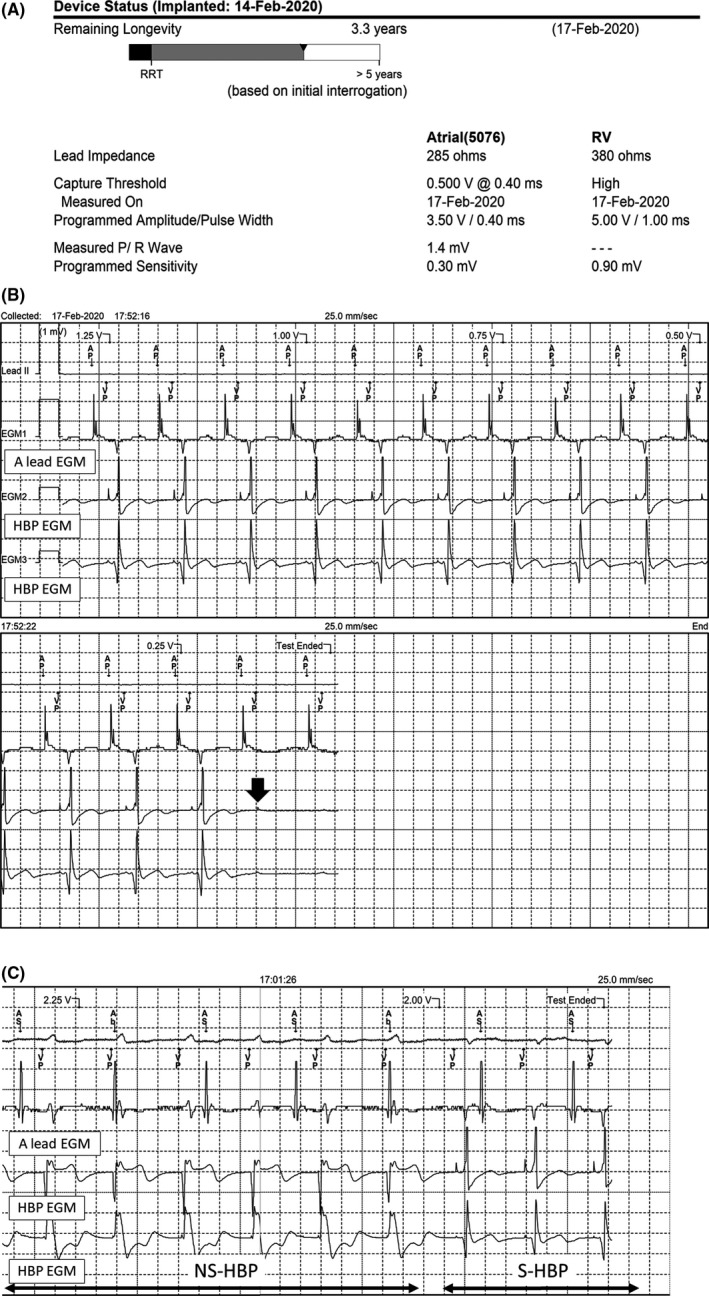
A, Device interrogation data on postoperative day 3. The automatic capture management (ACM) algorithm revealed a high ventricular capture threshold, and the ventricular lead pacing output was programmed to 5.00 V at 1.00 ms automatically. The estimated remaining generator longevity with the current device setting was 3.3 y. B, The His‐bundle pacing (HBP) lead threshold was tested manually during DDD pacing. Selective HBP with a pacing stimulus to the local ventricular electrogram time interval delay and pacing capture loss at 0.25 V at 0.4 ms (arrow). The ventricular capture management (VCM) algorithm performed a pacing threshold search through insertion of test paces and observation of the evoked response in the ventricle. For selective HBP, the VCM algorithm considers “pacing capture loss” because pacing stimulus evokes no immediate ventricular response. (C) With a pacing output higher than 2.25 V at 0.4 ms, nonselective HBP is noted with the culmination of both His‐bundle and ventricular capture, and the pacing QRS morphology is comparable different with that for selective HBP. In this case, the ACM algorithm overestimates the pacing threshold as 2.25 V at 0.4 ms

A 74‐year‐old patient with second‐degree atrioventricular block and hypertrophic cardiomyopathy presented with recurrent syncope. We arranged a permanent dual‐chamber pacemaker with HBP implantation for the patient. The patient was implanted with pacemaker leads on the His bundle (Medtronic 3830 lead) and on the right atrial appendage (Medtronic 5076 lead), and these were connected to a Medtronic Astra XT DR MRI SureScan generator. Selective HBP (S‐HBP) was successfully achieved with a minimal threshold of <1.5 V at 0.4 ms (Figure 1). Electrocardiogram (ECG) measurements obtained immediately after the operation revealed a sinus rhythm with S‐HBP. However, an ECG on postoperative day 1 revealed a sinus rhythm with nonselective HBP (NS‐HBP; Figure 2). The pacemaker interrogation report revealed a high HBP lead threshold, and the ventricular lead pacing output was programmed to 5.00 V at 1.00 ms automatically. The remaining longevity of the pacemaker was 3.3 years according to the current setting (Figure 3A). Chest radiographs revealed no evidence of lead dislodgement. In fact, the HBP lead threshold was 0.5 V at 0.4 ms according to a manual test (Figure 3B). Finally, we changed the ACM setting from “adaptive” to “monitor only” and fixed the pacing output to 3.5 V at 0.4 ms initially to avoid unnecessary battery depletion.

## 3 DISCUSSION

Automatic capture management is a programmable feature that allows automatic pacing output adjustment in response to pacing thresholds changes. Studies have indicated that automatic modulations of pacing output have been adjudicated appropriately in atrial, right ventricular, left ventricular, defibrillation, and epicardial leads.^5^, ^6^, ^7^ In our case, the HBP lead inserted into the right ventricular port and ACM use the ventricular capture management (VCM) algorithm. The Medtronic VCM algorithm consists of five steps to define a pacing threshold: (a) check for stable rhythm, (b) perform a pacing threshold search, (c) determine the capture using an evoked response, (d) determine the threshold, and (e) determine the strength‐duration curve for the final setting. Three VCM algorithm modes can be chosen including “adaptive,” “monitor only,” and“off.” In the adaptive mode, to maintain a safety margin, the VCM program pacing output is spontaneously regulated at a level 1.5‐ or 2‐fold of the measured threshold. In monitor‐only mode, the VCM program records only the pacing threshold data measurements and does not change the pacing parameters; moreover, it automatically performs a ventricular pacing threshold search through insertion of test paces and observation of the evoked response in the ventricle. Because of the time interval between pacing stimulation and the ventricular electrogram during HBP, the VCM algorithm considers “pacing capture loss” despite His‐bundle capture. The VCM algorithm increases pacing output until NS‐HBP with the culmination of both the His‐bundle and ventricular capture (Figure 3C). The VCM algorithm overestimates the accurate HBP threshold and unnecessarily changes pacing parameters to a high ventricular pacing output. Our recommended solution is programing the VCM from “adaptive” to “monitor only” such that no automatic adjustments occur.

His‐bundle pacing leads usually have a higher capture threshold than traditional RVP leads do, which may cause early battery depletion, necessitating early generator changes.^2^, ^8^ The default ACM algorithm in a Medtronic IPG is “adaptive,” with the program pacing output at twice the safety margin. Furthermore, experts suggest that changing the ACM algorithm from “adaptive” to “off or monitor” conserves battery life.^1^, ^3^

Because of the unique electrical parameters required for HBP, a dedicated His‐pacing port and specifically designed ACM algorithm for HBP should be considered in future IPG design.

## 4 CONCLUSION

This case report confirms that the ACM algorithm may overestimate the accurate HBP threshold and cause unnecessary battery depletion when using the default ACM algorithm of Medtronic IPGs. In addition, the HBP lead usually has a higher capture threshold than the traditional RVP lead does. A routine change in the ACM algorithm from “adaptive” to “off or monitor” is required for the conservation of device longevity.

## CONFLICT OF INTEREST

The authors declare no conflicts of interest.

## AUTHOR CONTRIBUTION

HPW: involved in the management of the case and wrote the manuscript. JYC and KHL: involved in revising the manuscript. KCC: involved in revising the manuscript and in final approval.

## WRITTEN CONSENT

Published with written consent of the patient.
